# Systemic sclerosis in children and older juvenile-onset patients: real-life map by the Egyptian college of Rheumatology

**DOI:** 10.1038/s41598-026-52795-y

**Published:** 2026-05-19

**Authors:** Hania S. Zayed, Tamer A. Gheita, Nevin Hammam, Manal Hassanien, Gehad Gamal Maghraby, Nermeen Samy, Nermeen N. Aziz, Abdelhfeez Moshrif, Yousra H. Abdel-Fattah, Amany R. El-Najjar, Enas A. Abdelaleem, Fatma A. Mohamed, Rania M. Gamal, Amany S. El-Bahnasawy, Khaled A. A. Abdelgalil, Dina F. El-Essawi, Reem El-Mallah, Thanaa F. Mansour, Eman F. Mohamed, Noha A. Azab

**Affiliations:** 1 Rheumatology Department, Faculty of Medicine, Cairo University, Kasr Alainy, Cairo, 11956 Egypt; 2 Rheumatology Department, Faculty of Medicine, Assiut University, Assiut University street, Assiut, 71515 Egypt; 3 Internal Medicine Department, Rheumatology and Clinical Immunology Unit, Faculty of Medicine, Cairo University, Kasr Alainy, Cairo, 11956 Egypt; 4 Internal Medicine Department, Rheumatology Unit, Faculty of Medicine, Ain Shams University, Abbasiya, Cairo, 11566 Egypt; 5 Rheumatology Department, Faculty of Medicine, Al- Azhar University, 1 Al-Azhar University Square, King Faisal, Assiut, 71524 Egypt; 6 Rheumatology Department, Faculty of Medicine, Alexandria University, Elazarita, Medan Elkhartoum, 21111 Alexandria Egypt; 7 Rheumatology Department, Faculty of Medicine, Zagazig University, Al Sharkia Governorate, Zagazig, 44519 Egypt; 8 Rheumatology Department, Faculty of Medicine, Beni- Suef University, Mokbel, Beni-Suef, 62511 Egypt; 9 Rheumatology Department, Faculty of Medicine, Minia University, Egypt-Aswan Agricultural Road, Minia, 61519 Egypt; 10 Rheumatology Department, Faculty of Medicine, Mansoura University, Elgomhoreya street, Mansoura, 35516 Egypt; 11 Rheumatology Department, Faculty of Medicine, Aswan University, Aswan, 81528 Egypt; 12 Health Radiation Research Department, Internal Medicine Unit, Rheumatology Clinic, National Centre for Radiation Research and Technology (NCRRT), Egyptian Atomic Energy Authority, 3 Ahmed Elzmor street, Nasr City, Cairo, 11762 Egypt; 13 Rheumatology Department, Faculty of Medicine, Ain Shams University, Abbasiya, Cairo, 11566 Egypt; 14 Internal Medicine Department, Rheumatology Unit, Faculty of Medicine, Tanta University, Elbahr Street, Tanta, 31111 Al Gharbiya Governorate Egypt; 15 Internal Medicine Department, Rheumatology Unit, Faculty of Medicine for Girls, Al-Azhar University, 38 Youssef Abbas street, Nasr City, Cairo, 11754 Egypt

**Keywords:** Children, Egyptian college of Rheumatology, Gender, Juvenile-onset, Overlap, Pediatric systemic sclerosis, Diseases, Medical research, Rheumatology

## Abstract

The aim of this work was to present the demographic and clinical features of children with juvenile systemic sclerosis (JSSc) compared to their older juvenile-onset (Jo-SSc) counterparts. The study included 12 JSSc children (< 16 years) and 54 Jo-SSc patients > 16 years (including adolescents and adults) recruited from 15 tertiary care centers across Egypt. Patients were classified as diffuse (dcSSc), limited cutaneous SSc (lcSSc) and overlap. The mean age at disease onset was 14 ± 2.5 years. Patients were 62 females and 4 males (F: M 15.5:1); 37 lcSSc, 26 dcSSc and 3 overlap. Disease features were generally comparable across five regions of Egypt. Pitting scars and hand puffiness were significantly more frequent in JSSc (58.3% and 41.7%) compared to Jo-SSc (16.7% and 14.8%) (*p* = 0.006 and *p* = 0.049, respectively) while pulmonary hypertension was more common in Jo-SSc (40.7% vs. 8.3%, *p* = 0.045). All males, currently with Jo-SSc, had gastrointestinal involvement versus *n* = 24 (38.7%) in females, *p* < 0.0001. To conclude, SSc in children and in those who grow into adulthood remains a rare disease. Pitting scars and hand puffiness were more frequent in children with JSSc while pulmonary hypertension became prominent as they grew. Children characteristics are special for each nation yet with similarities in some features.

## Introduction

Systemic sclerosis (SSc), a heterogeneous autoimmune connective tissue disease, is characterized by microvascular dysfunction, immune dysregulation, and progressive fibrosis of the skin and internal organs. Based on the extent of cutaneous involvement, SSc is commonly classified into limited cutaneous (lcSSc) and diffuse cutaneous (dcSSc) subtypes. Raynaud’s phenomenon is typically the earliest clinical manifestation, followed by musculoskeletal and internal organ involvement^1^.

Although rare in pediatrics, juvenile SSc (JSSc) is a severe and life-threatening condition that significantly impacts children’s development^2^ and physical function^3^. Epidemiological data indicate a very low incidence; the estimated prevalence in the USA is 3 in 1,000,000 children^4^. The annual incidence rate of JSSc in the UK and Ireland was 0.27 per million^5^ while it ranged from 0.98 to1.59 per million children in Japan^6^. The disease is more frequent in females, with reported female-to-male ratios of approximately 4:1^7^, and the mean age at diagnosis is between 8 and 12 years^8,9^.

Although JSSc has been considered less severe than adult-onset disease, largely due to lower rates of internal organ involvement, a distinct autoantibody profile, and better long-term outcome, cardiopulmonary involvement is the key determinant of outcome^8,10–12^. Clinical manifestations of organ involvement in JSSc usually evolve over time and can be modified by treatment, lifestyle factors, and comorbid conditions^8,9^.

During adulthood, juvenile onset SSc (Jo-SSc) patients were found to have similar disease subsets and pattern of organ involvement (lung, kidney, joint, muscle and heart) compared to adult-onset SSc. Although the overall autoantibody profile appears broadly similar, anticentromere antibodies (ACA) were less frequent in Jo-SSc (5%) than in adult-onset disease (26.9%)^13^.

Despite increasing recognition of JSSc, data from low- and middle-income regions remain limited^11^. Most available evidence derives from single-center cohorts or registry-based studies in high-income settings, which may not reflect disease expression in diverse healthcare contexts.

Given the rarity of JSSc and the need for long-term disease characterization, multicenter data from underrepresented regions are essential to improve understanding of clinical phenotypes and to inform future longitudinal research.

The aim of the present study was to compare the demographic features and disease characteristics between children with JSSc (age < 16 years) and their older Jo-SSc counterparts (> 16 years, including adolescents and adults) in order to help understand disease progression from childhood to adulthood.

## Methods

### Study design and participants

This multicenter cross-sectional study recruited patients with JSSc and Jo-SSc from 15 tertiary rheumatology centers across 5 Egyptian territories; Great Cairo (Capital), Alexandria, Delta, Canal cities and Upper Egypt between April 2024 and August 2025. The study was approved by the Ethical Committee at Assiut University [no 157300156] in accordance with the Declaration of Helsinki and patients provided an informed consent.

### Case definition

The current study included 66 SSc patients with disease onset before 18 years. Among those, there were 12 children with JSSc (< 16 years) and 54 Jo-SSc patients (> 16 years including adolescents and adults). The Jo-SSc patients were extracted from a nationwide SSc cohort including 1080 patients^14^. All patients fulfilled the Pediatric Rheumatology European Society/American College of Rheumatology/European League against Rheumatism (PRES/ACR/EULAR) classification criteria for JSSc^15^.

###  Data collection

A consistent excel sheet template was distributed and archived in an electronic database. Demographic data, SSc specific manifestations, laboratory tests and medications were collected during routine clinical visits and documented.

### Clinical definitions and organ assessment

Patients were classified as having dcSSc or lcSSc according to established criteria^16^. Skin involvement was evaluated using the modified Rodnan skin score (mRss)^17^ with documentation of Raynaud’s phenomenon, digital pitting scars or ulcers, hand puffiness, gangrene, microstomia, pigmentation changes and telangiectasia. Arthritis was recorded.

Pulmonary involvement was defined by the appearance of ground-glass opacities or fibrosis on high-resolution computed tomography (HRCT). Pulmonary function tests; forced vital capacity (FVC) and diffusing lung capacity for carbon monoxide (DLCO) were performed when available. After initial screening with echocardiography, pulmonary arterial hypertension (PAH) was diagnosed with right cardiac catheterization, which revealed an elevated mean pulmonary arterial pressure of ≥ 25 mmHg.

Cardiovascular involvement encompassed arrhythmias, conduction abnormalities, pericardial disease, or myocardial fibrosis as determined by clinical examination, electrocardiography or echocardiography. Gastrointestinal involvement included dysphagia and gastro-esophageal reflux disease, diarrhea or loss of weight. Renal involvement included scleroderma renal crises or ongoing renal damage due to SSc. Neurological involvement was defined as peripheral neuropathies or seizures associated with abnormal magnetic resonance imaging.

Laboratory investigations included complete blood count, erythrocyte sedimentation rate (ESR), C-reactive protein (CRP), serum creatinine, antinuclear antibodies (ANA), anti-double stranded DNA (anti-dsDNA), ACA, anti-topoisomerase I (anti-Scl-70) antibodies, and rheumatoid factor (RF). Double positivity refers to patients who tested positive for both anti-Scl-70 and ACA concurrently.

### Search strategy

The databases Scopus and Medline were searched for observational studies describing the clinical and serologic features of JSSc or Jo-SSc patients worldwide considering the more recent original articles including the largest number of SSc patients from each continent or region.

### Statistical analysis

Statistical Package for Social Science (SPSS) program version 25 was used for analysis of data. Data distribution was assessed using the Kolmogorov–Smirnov test. Data was presented as mean ± SD (range), number, and frequency (%) and missing data were adapted for. Mann–Whitney or Kruskal-Wallis tests were used for comparison of non-parametric continuous variables. The chi-square test was used for comparison of categorical variables between groups. Fisher’s exact test was applied when the expected frequency of any variable was less than 5. Significance was set at *p* < 0.05.

## Results

### Study population

The study included 66 patients with SSc, comprising 12 children with JSSc (< 16 years) and 54 Jo-SSc patients who were adults or adolescents aged between 16 and 18 years. The cohort consisted of 62 females and 4 males (F: M 15.5:1). Disease onset during late adolescence (age 17 years) was reported in 9 of 54 Jo-SSc patients. Characteristics of the studied patients are presented in Table 1.

**Table 1:** Characteristics of JSSc and Jo-SSc patients.

Parameters mean ± SD, *n*(%)	SSc patients (*n* = 66)			*p*
	All (*n* = 66)	JSSc < 16 years (*n* = 12)	Jo-SSc > 16 years (*n* = 54)	
Age (years)	21.8 ± 8.2	14.1 ± 1.9	23.6 ± 8.02	< 0.0001
Females	62 (93.9)	12 (100)	50 (92.6)	0.56
Disease duration (years)	7.8 ± 7.5	1.4 ± 0.74	9.3 ± 7.6	< 0.0001
Age at onset (years)	14 ± 2.5	12.8 ± 2.1	14.3 ± 2.5	0.041
Subtype: lcSSc: dcSSc	37:26	6:4	31:22	0.08
overlap	3 (4.5)	2 (16.7)	1 (1.9)	
mRss	11.5 ± 9	23.7 ± 8.3	13.7 ± 0.6	< 0.0001
Raynaud’s	30 (45.5)	7 (58.3)	23 (42.6)	0.25
Pitting scars	16 (24.2)	7 (58.3)	9 (16.7)	0.006
Digital ulcers	20 (30.3)	6 (50)	14 (25.9)	0.16
Hand puffiness	13 (19.7)	5 (41.7)	8 (14.8)	0.049
Microstomia	9 (13.6)	3 (25)	6 (11.1)	0.34
Hypo/hyper pigmentation	16 (24.2)	5 (41.7)	11 (20.3)	0.15
Telangiectasia	18 (27.3)	4 (33.3)	14 (25.9)	0.72
Arthritis	13 (19.7)	3 (25)	10 (18.5)	0.65
Pulmonary fibrosis	26 (39.4)	4 (33.3)	22 (40.7)	0.75
PAH	23(34.8)	1 (8.3)	22(40.7)	0.045
Cardiovascular	13 (19.7)	1 (8.3)	12 (22.2)	0.43
Gastrointestinal	28 (42.4)	2 (16.7)	26 (48.1)	0.058
Renal	2 (3)	0 (0)	2 (3.7)	-
Neurological	1 (1.5)	0 (0)	1 (1.9)	-
Hemoglobin (g/dl)	11.4 ± 1.2	11.8 ± 1.1	11.2 ± 1.3	0.11
TLC (x10^3^/mm^3^)	6.7 ± 2.4	7.3 ± 2.9	6.5 ± 2.3	0.4
Platelets (x10^3^/mm^3^)	296.4 ± 80.3	309.1 ± 77.4	291.8 ± 82	0.52
ESR (mm/1st hr)	34.7 ± 19.7	27.5 ± 20.4	37.2 ± 19.1	0.18
RF	3 (4.5)	1 (8.3)	2 (3.7)	0.61
ANA	43 (65.2)	10 (83.3)	33 (61.1)	0.19
Anti-dsDNA	3 (4.5)	1 (8.3)	2 (3.7)	0.46
Anti-Scl-70	25 (37.9)	7 (58.3)	18 (33.3)	0.19
ACA	16 (24.2)	3 (25)	13 (24.1)	1
Double positivity	9 (13.6)	3 (25)	6 (11.1)	0.35
Steroids	37 (56.1)	7 (58.3)	30 (55.6)	1
Calcium channel blocker	36 (54.5)	4 (33.3)	32 (59.3)	0.11
Pentoxyphylline	20 (30.3)	7 (58.3)	13 (24.1)	0.036
Sildenafil	8 (12.1)	3 (25)	5 (9.3)	0.16
Hydroxychloroquine	13 (19.7)	4 (33.3)	9 (16.7)	0.23
Methotrexate	30 (45.5)	7 (58.3)	23 (42.6)	0.36
Azathioprine	17 (25.8)	2 (16.7)	15 (27.8)	0.72
Leflunomide	2 (3)	0 (0)	2 (3.7)	-
Cyclophosphamide	6 (9.1)	1 (8.3)	5 (9.3)	1
Mycophenolate mofetil	14 (21.2)	4 (33.3)	10 (18.5)	0.26
Biologic	6 (9.1)	2 (16.7)	4 (7.4)	0.3

### Comparison between JSSc and Jo-SSc groups

Children with JSSc had a significantly higher frequency of digital pitting scars (*p* = 0.006) and hand puffiness (*p* = 0.049), but a lower frequency of PAH (*p* = 0.045) compared to those with Jo-SSc.

### Disease subtype distribution

The characteristics of the studied patients were compared according to the disease subtype (Table 2). Among the overall cohort, 37 patients (56.1%) had lcSSc, 26 (39.4%) dcSSc, and 3 (4.5%) had overlap syndromes. Overlap conditions included rheumatoid arthritis (RA) in one Jo-SSc patient and systemic lupus erythematosus (SLE) or polymyositis in two JSSc patients. Sjögren’s syndrome was present in two overlap cases. A family history of RA or SLE was reported in two Jo-SSc patients.

**Table 2:** Characteristics of the SSc patients according to subtype.

Parameters mean ± SD, *n*(%)	SSc subtypes (*n* = 66)			
	lcSSc (*n* = 37)	dcSSc (*n* = 26)	Overlap (*n* = 3)	*p*
Age (years)	20.1 ± 6	25 ± 10.1	15.3 ± 0.58	0.02
Females	36 (97.3)	23 (88.5)	3 (100)	0.32
Disease duration (years)	6.6 ± 5.1	10.4 ± 9.9	1.3 ± 0.57	0.04
Age at onset (years)	13.6 ± 2.5	14.7 ± 2.7	14	0.25
mRss	11.5 ± 9	23.7 ± 8.3	13.7 ± 0.6	< 0.0001
Raynaud’s	11 (29.7)	17 (65.4)	2 (66.7)	0.015
Pitting scars	4 (10.8)	11 (42.3)	1 (33.3)	0.015
Digital ulcers	6 (16.2)	12 (46.2)	2 (66.7)	0.015
Hand puffiness	7 (18.9)	4 (15.4)	2 (66.7)	0.11
Microstomia	1 (2.7)	6 (23.1)	2 (66.7)	0.002
Hypo/hyper pigmentation	7 (18.9)	7 (26.9)	2 (66.7)	0.16
Telangiectasia	9 (24.3)	8 (30.8)	1 (33.3)	0.83
Arthritis	4 (10.8)	6 (23.1)	3 (100)	0.001
Pulmonary fibrosis	10 (27)	14 (53.8)	2 (66.7)	0.06
PAH	14 (37.8)	7 (26.9)	2 (66.7)	0.33
Cardiovascular	7 (18.9)	6 (23.1)	0 (0)	0.63
Gastrointestinal	14 (37.8)	14 (53.8)	0 (0)	0.14
Renal	2 (5.4)	0 (0)	0 (0)	-
Neurological	1 (2.7)	0 (0)	0 (0)	-
Hemoglobin (g/dl)	11.6 ± 1.3	11.1 ± 1.2	11.7 ± 1.2	0.39
TLC (x10^3^/mm^3^)	6 ± 2.3	7.8 ± 2.3	4.7 ± 1.2	0.014
Platelets (x10^3^/mm^3^)	281.1 ± 52	321.9 ± 103.7	248 ± 53.7	0.15
ESR (mm/1st hr)	32 ± 20.5	36 ± 20	48 ± 1.7	0.41
RF	0 (0)	2 (7.7)	1 (33.3)	0.016
ANA	22 (59.5)	18 (69.2)	3 (100)	0.32
Anti-dsDNA	1 (2.7)	0 (0)	2 (66.7)	< 0.0001
Anti-Scl-70	13 (35.1)	9 (34.6)	3 (100)	0.08
ACA	11 (29.7)	2 (7.7)	3 (100)	0.001
Double positivity	4 (10.8)	2 (7.7)	3 (100)	< 0.0001
Steroids	19 (51.4)	15 (57.7)	3 (100)	0.26
Calcium channel blocker	19 (51.4)	15 (57.7)	2 (66.7)	0.86
Pentoxyphylline	10 (27)	7 (26.9)	3 (100)	0.03
Sildenafil	2 (5.4)	4 (15.4)	2 (66.7)	0.007
Hydroxychloroquine	7 (18.9)	3 (11.5)	3 (100)	0.001
Methotrexate	20 (54.1)	10 (38.5)	0 (0)	0.13
Azathioprine	13 (35.1)	4 (15.4)	0 (0)	0.12
Leflunomide	1 (2.7)	1 (3.8)	0 (0)	0.92
Cyclophosphamide	3 (8.1)	1 (3.8)	2 (66.7)	0.002
Mycophenolate mofetil	5 (13.5)	6 (23.1)	3 (100)	0.002
Biologic	0 (0)	3 (11.5)	3 (100)	< 0.0001

### Sex-related comparisons in Jo-SSc

In the Jo-SSc group, age at onset tended to be higher in males than females (15.8 ± 1.3 vs. 13.9 ± 2.6 years, *p* = 0.054). All 4 male patients had gastrointestinal involvement and were receiving calcium channel blockers; both were significantly more frequent than among females (*p* < 0.0001). None of the males was receiving methotrexate, sildenafil, cyclophosphamide, or biologics due to specific clinical considerations of this small group of patients. ACA was negative in all males. No other differences were observed between male and female Jo-SSc patients (results not shown).

### Treatment characteristics

Treatment strategies were determined by clinical manifestations. For Raynaud’s phenomenon, calcium channel blockers and pentoxyphilline were prescribed with addition of sildenafil in severe cases. Patients with interstitial lung disease were treated with cyclophosphamide, mycophenolate mofetil or azathioprine. PAH was managed with sildenafil. Hydroxychloroquine, methotrexate, leflunomide and small dose corticosteroids were used as a single treatment or in combination for the treatment of arthritis.

### Geographic distribution

Patients were recruited from multiple regions of Egypt, including Greater Cairo (*n* = 32), Alexandria (*n* = 1), the Delta (*n* = 7), Canal cities (*n* = 1), and Upper Egypt (*n* = 25). No remarkable differences in clinical characteristics were observed across regions.

## Discussion

JSSc is a rare disease, representing less than 10% of all SSc cases worldwide^11,14,18–20^, although a higher frequency has been reported in certain cohorts, such as in Zimbabwe^21^.

The present multicenter cohort provides important data from an underrepresented region where epidemiological and clinical characterization remains limited.

Consistent with previous international reports, the disease demonstrated marked female predominance and heterogeneous clinical manifestations. The female-to-male ratio observed in this study (15.5:1) was higher than that reported in the International Juvenile Scleroderma Inception Cohort (4:1)^7^, but comparable to findings from Japan (16:1)^19^ and Brazil (9.3:1)^20^. Such variation may reflect differences in genetic background or study design.

Different studies worldwide reported a mean age at disease onset of (10.3–12.7 years)^20,22–24^. In this work, the mean age at disease onset was slightly higher (14 ± 2.5 years) and similar to the study by Foeldvari et al.^25^. This difference may reflect variations in inclusion criteria, as patients with disease onset before 18 years were included. JSSc remains associated with significant morbidity, with complications that may substantially affect functional outcomes and survival^26^. Differences were observed in the disease characteristics between studies from different parts of the world, including the current one (Table 3)^2,3,7,10,11,22–24,26–31^.

**Table 3:** International stance of childhood SSc cases.

Parameters	EUROPE			ASIA					International		USA		South America		Egypt current work
	France ^2^	Poland ^3^	Italy ^30^	Turkey ^22^	China ^24^	Korea ^23^	Japan ^27^	India ^11^	PRES ^26^	IJSIC ^7^	CARRA ^29^	USA ^10^	Argentina ^28^	Mexico ^31^	
Number	18	22	18	25	64	13	16	23	134	175	64	111	23	15	12
Age at onset (*average year*)	10	6.8	7.8	10.3	11.4	11	8	-	9.5	10.4	10.3	10–16	6	7.6	12.8
Female: Male	8:1	2.5:1	2:1	7.3:1	1.9:1	1.6:1	1.3:1	4.8:1	4.6:1	4.3:1	5.4:1	4.8:1	10.5:1	6.5:1	12:0
lcSSc/dcSSc	1.25:1	0.25:1	0.2:1	0.32:1	0.83:1	0.75:1		0.64:1	0.1:1	0.37:1		1.02:1	0:1	1.5:1	1.5:1
Overlap	27.8%	31.8%	-	-	-	46.2%	-	-	-	17.3%	6.3%	29%	-	-	16.7%
Raynaud’s	72.2%	90.9%	61.1%	100%	73.1%	84.6%	80%	82.6%	86.6%	90%	73%	97%	83%	86.7%	58.3%
*Cutaneous*															
Pitting scars	-	-	-	-	-	-	-	-	-	-	-	-	65%	-	58.3%
Digital ulcers	11.1%	27.3%	-	64%	46.2%	53.8%	-	60.9%	-	53%	46%	-	-	46%	50%
Telangiectasia	50%	-	-	-	-	15.4%	56.3%	-	-	36%	36%	-	17.4%	46.7%	33.3%
*Organs involved*															
Arthritis	22.2	22.7%	55.6%	40%	57.7%	46.2%	43.8%	34.8%	32.1%	18%	19%	82%	35%	26.7%	25%
Pulmonary	77.8	72.7%	50%	48%	69.2%	53.8%	25%	65.2%	44%	34%	34%	55%	65%	73%	33.3%
Gastrointestinal	77.8	27.3%	83.3%	24%	38.5%	15.4%	20%	30.4%	31.3%	40%	42%	74%	39%	-	16.7%
Neurological	-	13.6%	-	-	7.7%	4%	-	-	2.2%	3%	-	-	4.3%	-	0%
Renal	0%	0%	-	8%	1.6%	7.7%	12.5%	0%	5.2%	4%	3%	4%	8.7%	-	0%
Cardiovascular	33.3%	22.7%	22.2%	-	21.6%	7.7%	18.8%	-	11.9%	6%	2%	17%	8.7%	-	8.3%
PAH	5.6%	9.1%	-	8%	11.5%	15.4%	-	4.3%	8.2%	5%	2%	-	8.7%	6.6%	8.3%
*Skin severity*															
mRss	11	18–40	10.4	18.4	12	-	-	22	-	12	-	-	-	-	14
*Autoantibodies*															
RF	-	22.7%	-	-	-	0%	37.5%	-	-	-	-	-	4.3%	-	8.3%
ANA	100%	86.7%	100%	92%	-	100%	75%	78.9%	-	91%	84%	97%	74%	-	83.3%
Anti-dsDNA	-	-	-	-	-	0%	0%	-	-	-	-	-	-	-	8.3%
Anti-Scl-70	44.4%	18.2%	22.2%	28%	55.8%	53.8%	31.3%	-	-	35%	46%	20%	8.7%	26.7%	58.3%
ACA	11.1%	31.8%	5.6%	-	-	0%	0%	0%	-	4%	15%	8%	4.3%	26.7%	25%

All children with JSSc in this study were female, whereas the Jo-SSc group included four males. Although male patients tended to have a higher age at disease onset and more frequent gastrointestinal involvement, these findings differ from those of Foeldvari et al.^7^ who did not demonstrate sex-related differences in onset age or frequency of gastrointestinal involvement. However, they reported a higher frequency of very low body mass index and more severe organ manifestations in male patients at presentation.

Nearly all patients with SSc have Raynaud’s phenomenon^1^, however, lower frequencies were observed among JSSc and Jo-SSc (58.3% and 42.6%, respectively), which may be attributable to documentation variability across centers or recall bias. Among the JSSc patients, nearly all patients with Raynaud’s phenomenon had pitting scars or digital ulcers reflecting differences in disease severity at presentation or referral bias.

The ratio of lcSSc: dcSSc (1.4:1) was comparable to that in Jo-SSc patients from the EUSTAR database^13^. Skin involvement, as assessed by mRss, among the Jo-SSc patients appeared less severe than in a study from Japan (16.4 ± 10.7 vs. 23.8 ± 12.7)^19^. Digital ulcers were also less frequent (25.9%) than in other reports (45.5%- 65%)^18–20^. While arthritis (18.5%) and pulmonary involvement (40.7%) in Jo-SSc patients were within previously described ranges^6,18–20,25^, PAH was much more frequent (40.7%) than in studies from Brazil (3.2%)^20^ and Japan (6%)^19^. The gastrointestinal tract was involved in nearly half of Jo-SSc patients, comparable to other studies^18,20^ but less frequent than in a study from Japan (65%)^19^. The cardiovascular system was involved in 22.2% of patients while renal and neurological involvement were rare, confirming previous observations^20^.

Autoantibody profiles showed variability across studies, further underscoring geographic heterogeneity in disease phenotype. Among the Jo-SSc patients, the frequency of ANA was 61.1% compared to 23% in a previous study^25^. Anti-Scl-70 were detected in one third of cases and ACA was positive in 24.1%, while the frequency of Anti-Scl-70 ranged from 22.6% to 88% and that of ACA ranged from 2% to 24% in other patient groups^6,19,20,25^.

Comparison between JSSc and Jo-SSc patients suggested that cutaneous manifestations such as pitting scars and hand puffiness were more prominent in children with JSSc, whereas PAH was more frequent among Jo-SSc cases. These findings may reflect disease evolution over time, consistent with a previous observation that JSSc clinical manifestations may progress during long-term follow-up^9^. No differences were observed in the antibody profile. In contrast, a Japanese nationwide survey compared JSSc under 20 years and adults with Jo-SSc over 20 years of age; Raynaud’s phenomenon, total renal involvements, ACA and U1RNP antibodies were significantly more frequent among adults while myositis was statistically more common in the young group^6^.

The predominance of lcSSc observed in this study aligns with findings from France and Mexico^2,31^, although diffuse disease predominates in other cohorts^19,32^. In this study, 56.1% had lcSSc, 39.4% dcSSc and 4.5% had overlap. Overlap syndrome was less frequent than in other studies (17% −29%)^6,10,32^. Patients with dcSSc had a higher mRss, more frequent Raynaud’s phenomenon and digital tip ulceration but the frequencies of pulmonary and cardiovascular manifestations were comparable between lcSSc and dcSSc. On the other hand, Foeldvari et al.^32^ reported similar frequencies of Raynaud’s phenomenon in both disease subtypes, a higher frequency of interstitial lung disease in dcSSc and more cardiac involvement in lcSSc patients. Patients with overlap in the current study had a significantly higher frequency of arthritis, a higher frequency of ACA and of double positivity of anti-Scl-70 and ACA compared to other disease subtypes. These results are in partial agreement with Foeldvari et al.^32^.

Overall, these findings reinforce the heterogeneity of JSSc across populations and highlight the need for standardized approaches to disease assessment. The small sample size, particularly among pediatric patients, and the cross-sectional design limit statistical power, generalizability of the observed associations, and the ability to draw definitive conclusions regarding disease trajectory. Given the exploratory nature of the analyses, adjustment for multiple testing was not performed. Variability in access to diagnostic investigations across centers may also have influenced the reported frequency of organ involvement.

In conclusion, this multicenter cohort provides valuable insight into JSSc and Jo-SSc from an underrepresented region. Although certain clinical features differ from previously reported cohorts, many findings are within established ranges. Larger prospective studies are required to better define disease evolution, regional variability, and long-term outcomes.

## Data Availability

The datasets used and/or analyzed during the current study are available from the corresponding author on reasonable request.

## Abbreviations

ACA: Anticentromere antibodies
ACR: American College of Rheumatology
ANA: Antinuclear antibodies
Anti-dsDNA: Anti-double stranded DNA
Anti-Scl-70: Anti-topoisomerase I antibodies
CARRA: Childhood Arthritis and Rheumatology Research Alliance
CRP: C-reactive protein
dcSSc: Diffuse cutaneous systemic sclerosis
DLCO: Diffusing lung capacity for carbon monoxide
ESR: Erythrocyte sedimentation rate
EULAR: European League against Rheumatism
FVC: Forced vital capacity
HRCT: High-resolution computed tomography
IJSIC: International Juvenile Scleroderma Inception Cohort
JSSc: Juvenile systemic sclerosis
Jo-SSc: Juvenile-onset systemic sclerosis
lcSSc: Limited cutaneous systemic sclerosis
mRss: Modified Rodnan skin score
PAH: Pulmonary arterial hypertension
PRES: Pediatric Rheumatology European Society
RA: Rheumatoid arthritis
RF: Rheumatoid factor
SLE: Systemic lupus erythematosus
SPSS: Statistical package for the social sciences
SSc: Systemic sclerosis
TLC: total leucocytic count
